# Taste Preference-Related Genetic Polymorphisms Modify Alcohol Consumption Behavior of the Hungarian General and Roma Populations

**DOI:** 10.3390/genes14030666

**Published:** 2023-03-07

**Authors:** Ali Abbas Mohammad Kurshed, Ferenc Vincze, Péter Pikó, Zsigmond Kósa, János Sándor, Róza Ádány, Judit Diószegi

**Affiliations:** 1 Department of Public Health and Epidemiology, Faculty of Medicine, University of Debrecen, 4028 Debrecen, Hungary; 2 Doctoral School of Health Sciences, University of Debrecen, 4032 Debrecen, Hungary; 3 Institute of Nutrition and Food Science, University of Dhaka, Dhaka 1000, Bangladesh; 4 ELKH-DE Public Health Research Group, University of Debrecen, 4028 Debrecen, Hungary; 5 Department of Health Methodology and Public Health, Faculty of Health Sciences, University of Debrecen, 4400 Nyíregyháza, Hungary; 6 Department of Public Health, Semmelweis University, 1089 Budapest, Hungary

**Keywords:** alcohol consumption, AUDIT, taste preference, genetic polymorphisms, genetic association, Hungarian population, Roma population

## Abstract

Harmful alcohol consumption has been considered a major public health issue globally, with the amounts of alcohol drunk being highest in the WHO European Region including Hungary. Alcohol consumption behaviors are complex human traits influenced by environmental factors and numerous genes. Beyond alcohol metabolization and neurotransmitter gene polymorphisms, taste preference-related genetic variants may also mediate alcohol consumption behaviors. Applying the Alcohol Use Disorders Identification Test (AUDIT) we aimed to elucidate the underlying genetic determinants of alcohol consumption patterns considering taste preference gene polymorphisms (*TAS1R3* rs307355, *TAS2R38* rs713598, *TAS2R19* rs10772420 and *CA6* rs2274333) in the Hungarian general (HG) and Roma (HR) populations. Alcohol consumption assessment was available for 410 HG and 387 HR individuals with 405 HG and 364 HR DNA samples being obtained for genotyping. No significant associations were found between *TAS1R3* rs307355, *TAS2R19* rs10772420, and *CA6* rs2274333 polymorphisms and alcohol consumption phenotypes. Significant associations were identified between TAS2R38 rs713598 and the number of standard drinks consumed in the HG sample (genotype GG negatively correlated with the number of standard drinks; coef: −0.136, *p* = 0.028) and the prevalence of having six or more drinks among Roma (a negative correlation was identified in the recessive model; genotype GG, coef: −0.170, *p* = 0.049), although, none of these findings passed the Bonferroni-corrected probability criterion (*p* > 0.05). Nevertheless, our findings may suggest that alcohol consumption is partially driven by genetically determined taste preferences in our study populations. Further studies are required to strengthen the findings and to understand the drivers of alcohol consumption behavior in more depth.

## 1. Introduction

Hazardous drinking is a significant public health problem contributing to the development of more than 200 diseases and injuries [1] and resulting in 1.78 million deaths in 2020 worldwide [2]. In addition, among people 25–49 years of age, alcohol use was found to be the most important risk factor at the global level [3]. Among the many health and public health challenges of the COVID-19 crisis, an increased burden of alcohol consumption evolved during the pandemic situation [4]. Alcohol-related problems do not only arise at the individual level, but harm to others is also considered a substantial problem [1]. Alcohol-related disease burden affects populations disproportionally, the European Region together with Hungary being the most heavily affected [5]. Although alcohol consumption showed a decreasing trend in Hungary [5], in 2019 consumption levels were still above the OECD average and the country was among those nations, which reported consumption over 11 L (calculated for pure alcohol) per capita per year [6]. Furthermore, heavy alcohol use, alcohol use disorder, and dependence are still considered of public health significance [7] (alcohol use disorders: Hungary 21.2% vs. Europe 8.8%; dependence: Hungary 9.4% vs. Europe 3.7%) [5] and Hungary can be characterized with the highest standardized rates for alcohol-attributable mortality in Europe [8]. 

Alcohol consumption patterns and related harm vary not only across countries but also within the same country among ethnic groups [9], including the most disadvantaged Roma population of Europe and Hungary [10,11,12,13,14,15,16,17,18,19,20,21,22]. This minority population is mainly accumulated in Central and Eastern Europe [23] with a representation of over 5% of the total population [24] (8.9% of the total population, 876,000 individuals in 2013 in Hungary [25]) and faces discrimination, several barriers when seeking healthcare services, and poorer health outcomes compared to mainstream populations [26,27,28,29,30,31,32,33].

Alcohol use disorder (AUD) being a complex human trait shows a moderate heritability estimate of 0.49, though familial aggregation may also be due to shared environmental effects [34]. AUD and other alcohol consumption phenotypes are considered distinct but related phenotypes [35] with some overlap of genetic background [36]. These alcohol-related phenotypes can be considered as quantitative traits often measured by varying phenotype assessment methods [35] and are influenced by numerous genetic polymorphisms. 

The most extensively studied genetic variants regarding AUD and alcohol consumption are involved in the breakdown of alcohol (alcohol and aldehyde dehydrogenase-related genes and variants -*ADH* and *ALDH*), especially certain *ADH1B* and *ALDH2* polymorphisms showing the largest effects in Asian populations. Furthermore, polymorphisms of several neurotransmitter-related genes affected by alcohol (i.e., receptors, enzymes, and solute carriers of the cholinergic, dopamine, GABA, serotonin, glutamate, and opioid pathways) have been also subject to several studies [35]. 

Research suggests that oral sensations evoked by consumed beverages may also determine food and alcohol preferences and intake [37,38]. Although five basic taste qualities (bitter, sweet, sour, salty, umami) and the recently identified fat taste exist, bitter and sweet sensitivity has been found to influence alcohol consumption and preferences, though methodological difficulties are not easy to overcome when summarizing the results. Several studies rely on quinine bitterness as a measure for bitterness and others suggest PROP (6-n-propylthiouracil) taster status to be used for orosensory responsiveness for bitter and also use it as a marker for bitter sensitivity and preference. Several research groups found associations between PROP responsiveness and alcohol consumption behaviors [37,38]. Furthermore, wine/alcohol bitterness was also found to be associated with *TAS2R38* genetic variants, which has been widely investigated in relation to bitter and sweet taste preferences [38]. It was also shown that sweet-likers may be at an enhanced risk for the development of alcohol use disorders, which may be in connection with the sugar content of alcoholic beverages associated with the human neural reward system [37].

Although less extensively studied compared to alcohol metabolizing gene polymorphism and genetic variants related to neurotransmitters, whose levels are altered through drinking of alcohol, taste preference-related genetic variants may also influence alcohol consumption patterns. As investigated in the literature, most of the studies focus on *TAS2R38* variants rs713598, rs1726866 and rs10246939 [39,40,41,42,43,44,45,46,47,48,49,50,51,52,53] followed by mainly other *TAS2R* gene [43,45,46,54,55] and gustducin (*CA6*) variants [45,48]. Although the results related to single nucleotide polymorphisms (SNPs) of taste preference genes and alcohol consumption patterns were found to be conflicting and/or the number of studies in the literature was scarce [56], it may be still hypothesized that genetic polymorphisms related to bitter and sweet taste preferences may mediate alcohol consumption patterns in some way.

The number of genetic association studies investigating alcohol consumption behaviors of Roma communities in comparison with the relevant mainstream populations of different countries is also very limited. One study in Hungary found the *ADH1B* rs1229984 (carrying the ADH1B*2 allele) to decrease drinking frequency, furthermore, it was associated with lower odds for having more positive answers on the CAGE screening tool (Cut-down, Annoyed, Guilty, Eye-opener) and also for positive CAGE screening status [57]. In addition, the 272Gln/35Val allele (*ADH1C* rs1693482/rs698) homozygosity was demonstrated to increase the risk of excessive and problem drinking among men aged 45–64 years [58]. Another study analyzing the distribution and combined effect of alcohol metabolism and neurotransmitter gene polymorphisms in the general Hungarian and Roma populations found no over-representation of genetic alterations predisposing to alcohol dependence and lower genetic risk scores in the minority population [59]. Furthermore, Hubáček et al. identified *ADH1B* rs1229984 genotype frequencies in the Czech Roma population corresponding with frequencies of North India/Central Asia [60]. On the other hand, alcohol consumption phenotypes in relation to taste preference-related genetic variants in Roma populations in comparison with majority populations have not been investigated before [56]. 

The majority of genetic association studies investigating taste preference gene polymorphisms focused on drinking frequency and/or quantity [56]. Among these, the only study, which characterized alcohol consumption with the Alcohol Use Disorders Identification Test (AUDIT) used the first three questions of the questionnaire [46]. In our past work we characterized the alcohol consumption patterns of HG and HR populations and found no differences in risky alcohol use based on the AUDIT total scores between Roma and non-Roma [61]. Therefore, our present work aimed to elucidate the underlying genetic determinants of alcohol consumption patterns considering taste preference genetic variants in the Hungarian general (HG) and Roma (HR) populations using the first three questions of the AUDIT.

The potential genetically determined taste-driven preferences behind Hungary’s alcohol consumption levels should be considered when targeting alcohol-related harm considering also the possible ethnic-specific effect of these variants.

## 2. Materials and Methods

### 2.1. Study Design

Data collection was implemented in mid-2018 within the framework of a three pillar-based) comparative health survey involving physical examinations, blood sample collection, and questionnaire surveys [62]. Sampling of the study populations was based on the pre-set principle that if someone was unavailable to be reached, it was then allowed to include another individual, but it was not permissible to recruit another subject in place of someone, who refused to participate in the study. It was planned to include 500 people in both study samples. Practice nurses took the questionnaires of the survey in the Hungarian general population, meanwhile among Roma, this work was assigned to Roma university students. Blood samples were taken in General Practitioners’ (GPs) offices for subsequent genetic analysis. 

#### 2.1.1. Sample Representative of the Hungarian General Population of Northeast Hungary

The Hungarian reference sample was obtained from a population-based registry. This program, called the General Practitioners’ Morbidity Sentinel Stations Programme (GPMSSP) has been operating since 1998 to monitor the incidence and prevalence of chronic non-communicable diseases of great public health importance. The source population of this registry encompasses all individuals belonging to the practices of the 59 general practitioners (GPs) participating in the program [63,64]. Individuals in our study were randomly drawn from the GPMSSP, who were 20–64 years of age, not institutional residents and were registered by the participating primary care providers of Borsod-Abaúj-Zemplén and Szabolcs-Szatmár-Bereg counties of the northeastern part of the country. Based on the study design 25 subjects from each 20 randomly selected GP practices were to be involved in our research. Due to the refusal of two GPs, 450 subjects from the practices of the remaining (18) GPs were available in the final sample. Health behavior surveys were conducted during a health visit in the GPs’ practices by practice nurses.

#### 2.1.2. Sample Representative of Hungarian Roma of Northeast Hungary Living in Segregated Colonies

Participants of Roma segregated colonies from the same two counties of Northeast Hungary (Hajdú-Bihar and Szabolcs-Szamár-Bereg counties) were enrolled by a stratified multistep random method. Prior to this research, during a previously conducted environmental survey, segregated colonies, where the population size exceeded 100 individuals, were identified. The ethnicity of inhabitants in this investigation was confirmed by self-declaration [65]. After the validation of the colony registry database, 20 colonies were randomly chosen, and subsequently from each colony 25 households were randomly drawn. One person aged 20–64 years was enrolled by using a random table from each household yielding 500 subjects in the sample. Interviews were delivered by Roma university students, who had previously received appropriate training.

### 2.2. Alcohol Consumption Behavior Assessment

Alcohol consumption was assessed with the AUDIT questionnaire. The impact of SNPs in taste preference genes was evaluated on responses to the first three questions from this screening tool: “How often do you have a drink containing alcohol?”; “How many drinks containing alcohol do you have on a typical day when you are drinking?”; and “How often do you have six or more drinks on one occasion?” [46]. The AUDIT was provided in an interview version.

### 2.3. Selection of Single Nucleotide Polymorphisms

Systematic literature search was carried out in order to identify the most relevant single nucleotide polymorphisms (SNPs) related to taste preference genes, which may influence alcohol consumption behavior [56]. Based on this search, those polymorphisms were selected to include in this study, whose effect had been extensively studied in relation to bitter and sweet taste preference/perception [38] and may also be relevant when investigating alcohol consumption [56]. Altogether four SNPs were selected: *TAS1R3* rs307355, *TAS2R38* rs713598, *TAS2R19* rs10772420, and *CA6* rs2274333. The effect of these variants on alcohol consumption behaviors and taste-related phenotypes is summarized in Table 1.

### 2.4. DNA Preparation and Genotype Assessment

DNA isolation was performed using the MagNA Pure LC DNA Isolation Kit—Large Volume (Roche Diagnostics, Mannheim, Germany) following the manufacturer’s instructions, for which 500-μL aliquots of EDTA-anticoagulated blood samples were prepared. Extracted DNA samples were eluted in 200 μL MagNA Pure LC DNA Isolation Kit-Large Volume Elution Buffer. The Mutation Analysis Core Facility (MAF) of Clinical Research Center, Karolinska University Hospital (Stockholm, Sweden) provided the genotyping of SNPs of interest (and quality control) applying the Mass Array platform with iPLEX Gold Chemistry [104]. Successful genotyping rate exceeded 98 percent.

### 2.5. Statistical Analysis

Data analysis was carried out using the STATA 10.0 Statistical software (StataCorp LP, College Station, TX, USA). Comparison of sociodemographic characteristics and alcohol consumption frequencies were evaluated by chi-square and Fisher’s exact tests. Hardy–Weinberg equilibrium (HWE) was estimated using “hwsnp” [105] and allele frequencies by “genhw” [106] function in STATA. To test the significance of differences in the allele and genotype frequencies between the two samples the chi-square test was applied. The association between the first three questions of the AUDIT questionnaire and selected taste preference genetic polymorphisms was conducted by using STATA’s “qtlsnp” command [105,107] following dominant and recessive models, which were defined according to minor alleles (covariates: gender, age, marital status) in HG and HR populations separately yielding nominal *p*-values. Nominally significant *p*-values (<0.05) of the initial analyses were Bonferroni-corrected as well, since each SNP was tested for multiple associations in the two sample populations, in which the nominal *p*-values were multiplied by the total number of tests performed. Aggregate effect of genetic polymorphisms on alcohol consumption phenotypes was analyzed by summing the number of minor alleles of all four polymorphisms, i.e., calculating the unweighted genetic risk score. 

## 3. Results

Alcohol consumption assessment was available for 410 HG and 387 HR individuals, and 405 HG and 364 HR DNA samples were available for genotyping, respectively. No significant differences were found regarding the mean age of the two study populations (HG: 44.3 ± 12.3 years, HR: 42.8 ± 12.1 years, *p* = 0.075). The proportion of men was significantly lower among Roma than in the reference general population sample (0.26 vs. 0.44, *p* < 0.001). Being Roma was associated with lower educational attainment, higher unemployment rate, and less favorable self-perceived financial status (*p* < 0.001) but not with marital status (*p* = 0.240) according to the chi-square test. Further details on study population characteristics are summarized in Table S1. Analysis of drinking categories of the two populations according to the 1st three questions of the AUDIT questionnaire indicate that Roma consume alcohol less frequently (the crude frequency of 2–3 times a week or more was significantly lower in the HR sample (5.47%), than in the general one (12.75%)), but no other differences were observed (Table S2).

### 3.1. Allele and Genotype Comparisons between the Study Populations

Selected SNPs did not deviate significantly from the Hardy–Weinberg equilibrium in our study populations (Table S3). The genotype and allele frequencies (Table 2 and Table S4) did not show significant differences (*p* > 0.05) when comparing the two study samples. 

### 3.2. Association of SNPs with Alcohol Consumption Phenotypes

In our present study we could not identify significant associations between *TAS1R3* rs307355, *TAS2R19* rs10772420, and *CA6* rs2274333 polymorphisms and the alcohol consumption phenotypes analyzed. In the initial analyses significant associations were found between *TAS2R38* rs713598 and the number of standard drinks containing alcohol consumed in the HG sample. Among Roma, *TAS2R38* rs713598 predicted the prevalence of having six or more drinks on one occasion. However, none of these findings passed the Bonferroni-corrected probability criterion. All results of the association analyses are presented in Table S5. Significant associations of the initial analyses are depicted in more detail in Table 3.

#### 3.2.1. Sample Representative of the Hungarian General Population

The nonsynonymous coding *TAS2R38* (rs713598, Ala49Pro) SNP was observed to influence the number of standard drinks containing alcohol in the recessive model. Genotype GG negatively correlated with the number of standard drinks (coef: −0.136, *p* = 0.028, Table 3). However, after correcting for multiple comparisons applying the Bonferroni method, this result did not remain significant. 

#### 3.2.2. Sample Representative of Hungarian Roma Living in Segregated Colonies

A significant association was identified between the variant rs713598 of *TAS2R38* and the prevalence of having six or more drinks on one occasion. Similar to the HG sample regarding the number of standard drinks, a negative correlation was identified in the recessive model (genotype GG, coef: −0.170, *p* = 0.049, Table 3) albeit, this association did not persist after the Bonferroni correction.

### 3.3. Aggregated Effect of SNPs on Alcohol Consumption Phenotypes

Summation of the number of minor alleles of the four polymorphisms included in our study did not show any significant association (*p* > 0.05) with either of the alcohol consumption phenotypes in either of the study samples.

## 4. Discussion

This study aimed to explore possible associations of the most relevant taste preference-related genetic variants with alcohol consumption behavior in the Hungarian general and Roma populations. To our knowledge this is the first research investigating the effect of these genetic variants on drinking patterns in the Roma population in comparison with the mainstream population.

Our results indicate *TAS2R38* rs713598 have an impact on two different quantitative measures of alcohol consumption (number of drinks consumed and frequency of heavy drinking, respectively) in the HG and HR groups. This variant is the most extensively studied when considering bitter and even sweet taste preferences [38]. The variant rs713598 is one of the three functional variants located at the TAS2R38 locus (rs713598, rs1726866, rs10246939) determining certain bitter-tasting phenotypes. Regarding the PROP supertaster–taster–non-taster categories, by location, rs713598 (P/A) is the first one, rs1726866 (A/V) the second, and rs1024693 (V/I) the third. According to this, PAV (proline–alanine–valine) homozygotes can be characterized as tasters, which is also considered the dominant haplotype. AVI (alanine–valine–isoleucine) homozygotes are considered as insensitive (non-tasters) when considering the ability to taste such bitter substances. Heterozygotes possess moderate sensitivity to PROP and PTC (phenylthiocarbamide). It was demonstrated that rs713598 holds the greatest impact on bitter taste signal transduction, while rs1726866 holds less prominent effects, and rs10246939 polymorphism has eventually no detectable [108,109] effect at all. 

When searching the literature regarding the association of taste preference-related gene polymorphisms and alcohol consumption phenotypes, similar to bitter taste perception and preference, *TAS2R38* variants (rs713598, rs1726866, rs10246939) have been mainly investigated. However, the findings on the effect of these variants are contradictory [56]. Some of the findings, but not all, may suggest that individuals with taster genotypes/haplotypes consume less alcohol, however phenotype assessment methods and study populations vary widely among studies [56], although several studies failed to find an association between these genetic variants and alcohol consumption phenotypes [56]. In contrast to the studies, where individuals with higher bitterness perception are less likely to consume alcohol (the only study using the first three questions from AUDIT as measures for alcohol consumption, also found the major “C” allele to decrease alcohol consumption, although not in a general population sample but in a head and neck cancer cohort), our results suggest negative correlations (at the nominal significance level) in the recessive model (defined according to minor allele), indicating that individuals with genotype GG (non-tasters) consume less alcohol. In line with our results, one study reported similar findings, i.e., tasters consuming more alcohol, while also indicating this was not necessarily inconsistent with other research findings. This research suggests that there could be other factors promoting alcohol consumption among subjects with an enhanced ability to taste bitterness frequency [45], e.g., wine consumption may be associated with increased PROP bitterness perception [110,111]. Similar factors may explain our findings as well, since AUDIT results do not differentiate between drinks having different taste profiles.

The aforementioned findings showed an ethnicity-dependent pattern in our study in some way. In the HR population this variant was associated with the number of standard drinks consumed and among Roma with the prevalence of having six or more drinks on one occasion. Both questions refer to quantitative measures of alcohol consumption although from a slightly different approach. One potential reason for this finding could be that taste perception and preference may influence alcohol consumption differently in these populations in some respects. Furthermore, it is also possible that the taste profile of alcohol consumed by subjects in the two study samples also differed. From the genetic point of view other possible explanations also exist. According to the literature, ethnic specific findings were observed in several genetic association studies [112,113,114,115,116,117,118,119,120,121,122], even when considering taste preference-related genetic polymorphisms [123]. The reasons behind this phenomenon may be related to some ethnic variation in linkage disequilibrium (LD) [116,118], where the effect of genetic polymorphisms under investigation could be linked to other real predisposing genetic variants showing different strengths for associations across ethnic groups [119]. So, the effect of the investigated genetic variants might be diluted or masked by other sometimes even yet unidentified susceptibility genes, actually being responsible for the development of phenotypes of interest [114]. It cannot be excluded also that certain alleles act differently in certain populations [118,119]. Considering different alcohol consumption-related phenotypes it should be noted that these various phenotypes may encompass different genetic backgrounds.

In our present study no significant associations were observed for *TAS1R3* rs307355, *CA6* rs2274333, and *TAS2R19* rs10772420 polymorphisms. The rs307355 polymorphism of *TAS1R3* was found previously to influence taste sensitivity to sucrose (“T” alleles indicating reduced sensitivity) [38] and one study identified this variant as predicting some alcohol consumption-related phenotypes [43]. This variant is located in the 5′UTR (untranslated) region of *TAS1R3*, and leads to a cytosine to thymine substitution, potentially influencing the function of the regulatory element and gene transcription [66], and contributes to alterations in sweet and alcohol perception, although this relationship was not verified in our present study. 

The research on carbonic anhydrase VI (also called gustin), a zinc-metalloprotein, which is secreted by the salivary glands [124,125] also suggests it to be a trophic factor for the development and growth of taste buds [126]. The variant itself leads to an amino acid substitution (Ser90Gly) and is supposed to influence also the formation and function of fungiform papillae on the anterior tongue surface [127], potentially having an effect on PROP sensitivity. Although investigated by several research groups, the *CA6* rs2274333 yielded equivocal findings considering bitter taste preference [38] showed no correlation with alcohol consumption [56], which is in line with our findings.

The *TAS2R19* rs10772420 variant, which codes for an arginine-to-cysteine substitution at amino acid 299, was previously shown to be associated with the preference, intensity, detection threshold of bitter tasting compounds and preference of grape-fruit juice [38], although the possibility was raised that these findings may be due to strong LD between *TAS2R19* and nearby *TAS2R* genes [100,128]. Supporting our findings, none of the studies investigating the relationship of this variant on various alcohol consumption phenotypes could identify any association with consumption patterns [56].

Due to the limited findings in the literature, and the different measures of alcohol consumption used [35], additional investigations should be carried out to further explore the effect of these polymorphisms on alcohol consumption behaviors. 

Several potential limitations need to be recognized when interpreting the results of our study. 

Human subjects perceive alcoholic beverages as a combination of sweet and bitter tastes [129] with certain beverages having different taste profiles [130], although the main taste modality of alcohol consumed has not been investigated in our research, which may impact our findings. Furthermore, alcohol consumption is a subject liable to underreporting [131] even if it is estimated by using the AUDIT tool [132]. In addition, the effectiveness of the AUDIT tool may vary in some ethnic groups/minorities [133]. Research also suggests that Roma people may be more prone to please research investigators more than individuals of mainstream populations, potentially influencing questionnaire results [134,135,136]. Furthermore, the AUDIT was provided in an interview version in our study. Roma people are already subject to negative stereotypes, which may influence their answers on alcohol consumption, and they may rather answer questions in a manner that will be not viewed unfavorably by others. The potential difficulties in understanding the questions of the AUDIT, were addressed by having Roma students as interviewers. In addition, the AUDIT does not provide a comprehensive view on lifetime alcohol use and problems, and no information is available on the underlying causes behind abstinence, which could even be attributed to self-decision due to alcohol dependence [46].

Other possible limitations of our study could be attributed to characteristics of the Roma study population, which was not representative of the country’s overall Roma population, only representative of Roma in Northeast Hungary (where Roma are mainly accumulated), who live in segregated colonies. Ethnicity was based on self-declaration and since some Roma may be unwilling to self-report Roma ethnicity [137], the HG sample may therefore have also included some Roma individuals. In addition, only subjects aged 20–64 years were enrolled in our study. Individuals older than 64 were not considered, since in our previous Roma surveys the representation of persons aged 65 years and older was as low as 3–4% [33,138,139]. Furthermore, the representation of women among Roma was higher than that of the non-Roma population. This is in line with our previous surveys in similar settings of the country [139] and also to a cross-sectional study conducted in Slovakia [140]. This may be in connection with the time of data collection, which occurred during the day, when in most Roma households in this region (Northeast Hungary) women resided at home, while men travelled for public work [62].

## 5. Conclusions

Alcohol consumption is a complex trait influenced by numerous genes. This research is the first comparative study investigating the potential associations of taste preference-related genetic polymorphisms with drinking behaviors of the Hungarian general and Roma populations. We observed some ethnicity-specific associations between genetic variations in the *TAS2R38* receptor genes and certain aspects of alcohol consumption. Nevertheless, our initial results did not remain significant after correcting for multiple testing, but still our findings should be considered interesting [46,141,142] in raising the idea that alcohol consumption may be partially driven by genetically determined taste preferences in our study populations. Additional research is essential to replicate these findings, which could contribute to the better understanding of the drivers of alcohol consumption behavior in more depth. 

## Figures and Tables

**Table 1:** Effect of selected genetic polymorphisms on alcohol consumption behavior and taste phenotypes.

Gene	SNP	Association	No Association	Relation to Taste Phenotypes
*TAS1R3*	rs307355	Soju intake and heavy drinking (≥30 g/day; CT carriers more likely to be heavy drinkers) [43].	Wine, spirit, beer consumption [43].	Taste sensitivity to sucrose (reduced sensitivity associated with T alleles) [66].
*TAS2R38*	rs713598	Daily number of standard drinks (P allele carriers: fewer standard drinks, also from spirits and mixed drinks) [44]. Alcohol consumption frequency (tasters: higher frequency) [45]. Decreased alcohol consumption (C allele; first question of AUDIT [46].	Beer and wine consumption [44]. Second and third items of the AUDIT questionnaire [46]. Weekly alcohol consumption [42].	PTC, PROP, thioamide and salicin threshold, taster status, bitterness; preference for bitter vegetables (lower preference, threshold for tasters) [67,68,69,70,71,72,73,74,75,76,77]. Preference/threshold of sucrose, preference, and intake of sweet tasting foods (GG lower preference) [78,79,80].
*TAS2R38*	rs713598, rs1726866, rs10246939	More frequent and more alcohol consumption of AVI/AVI homozygotes [52,53]. Subjects with the positive association of AVI/AVI and being alcoholic [39]. Higher frequency of AVV homozygotes among alcohol consumers and association with increased alcohol intake [40]. Taster haplotype associated with a lower mean of the largest number of drinks (ever having in 24 h) [41] and lower weekly alcohol intake for subjects with at least one PAV haplotype [42]. AVI haplotypes were less likely to be alcohol consumers [43].	Daily alcohol consumption [47,48]. Beer and total daily alcohol consumption [43]. Alcohol drinker status [49,50]. Alcohol consumption frequency and amount [51].	PROP phenotype, bitterness of ethanol, cruciferous vegetable preference, intake (lower preference, threshold for tasters) [68,69,70,71,75,77,81,82,83,84,85,86,87,88,89,90,91,92,93,94,95,96]. Preference and intake of sweet tasting foods (PAV higher preference) [97,98].
*TAS2R19*	rs10772420	--	Alcohol consumption frequency [45]. First three items of the AUDIT questionnaire [46]. Drinking frequency and heavy drinker status [55].	Preference, intensity, detection threshold of bitter tasting compounds and preference of grape-fruit juice (The A allele was associated with more intense quinine perception) [53,99,100,101,102].
*CA6*	rs2274333	--	Daily consumption of alcohol [48]. Alcohol consumption frequency [45].	PROP (bitter) taster status, threshold (The A allele more common in supertasters) [82,84,86,103].

SNP: single nucleotide polymorphism; PROP: 6-n-propylthiouracil; PTC: phenylthiocarbamide; *TAS1R3*: Taste receptor type 1 member 3; *TAS2R38*: Taste 2 receptor member 38; PAV: Proline–alanine–valine (taster); AVI: Alanine–valine–isoleucine (non-taster); AVV: Alanine–valine–valine; P (C) allele: taster; *TAS2R19*: Taste receptor type 2 member 19; *CA6:* Carbonic anhydrase VI; AUDIT: Alcohol Use Disorders Identification Test.

**Table 2:** Genotype frequencies of selected genetic polymorphisms in the Hungarian general and Roma populations.

Gene	SNP	Genotypes	HG Genotype Frequency % (*n*)	HR Genotype Frequency % (*n*)	*p*-Value
		CC	81.2 (329)	82.7 (301)	
*TAS1R3*	rs307355	TC	17.8 (72)	16.5 (60)	0.864
		TT	1.0 (4)	0.8 (3)	
		CC	34.1 (136)	37.1 (134)	
*TAS2R38*	rs713598	GC	43.6 (174)	45.7 (165)	0.203
		GG	22.3 (89)	17.2 (62)	
		AA	19.3 (78)	16.4 (59)	
*TAS2R19*	rs10772420	AG	49.6 (201)	46.8 (169)	0.215
		GG	31.1 (126)	36.8 (133)	
		AA	50.0 (199)	50.3 (182)	
*CA6*	rs2274333	AG	38.9 (155)	41.1 (149)	0.490
		GG	11.1 (44)	8.6 (31)	

SNP: single nucleotide polymorphism; HG: Hungarian general population; HR: Hungarian Roma population; *TAS1R3*: Taste receptor type 1 member 3; *TAS2R38*: Taste 2 receptor member 38; *TAS2R19*: Taste receptor type 2 member 19; *CA6*: Carbonic anhydrase VI; Values are presented as % (*n*).

**Table 3:** Association of selected genetic polymorphisms with alcohol consumption behavior in the Hungarian general and Roma populations.

Gene, SNP	Phenotype	Population	Genetic Model	Reference	Genotype	Coef	*p*-Value	
*TAS2R38* rs713598	AUDIT2	HG	Recessive	CC or GC	GG	−0.136	0.028 (0.224 *)	
*TAS2R38* rs713598	AUDIT3	HR	Recessive	CC or GC	GG	−0.170	0.049 (0.392 *)	

SNP: single nucleotide polymorphism, HG: Hungarian general population; HR: Hungarian Roma population; *TAS2R38*: Taste 2 receptor member 38; AUDIT2: “How many standard drinks containing alcohol do you have on a typical day when drinking?” (0p: 1 or 2; 1p: 3 or 4; 2p: 5 or 6; 3p: 7 to 9; 4p:10 or more); AUDIT3: “How often do you have six or more drinks on one occasion?” (0p: Never; 1p: Less than monthly; 2p: Monthly; 3p: Weekly; 4p: Daily or almost daily); Models were defined according to minor alleles. Coef: regression coefficient; Covariates: age, gender, marital status; only at least nominally significant results are presented in this table. * Bonferroni-corrected *p*-values.

## Data Availability

Not applicable.

## Supplementary Materials

The following supporting information can be downloaded at: https://www.mdpi.com/article/10.3390/genes14030666/s1, Table S1: Demographic characteristics of the two study samples; Table S2: Drinking categories of the Hungarian general and Roma populations according to the 1st three questions of the AUDIT questionnaire; Table S3: Hardy–Weinberg equilibrium test for selected genetic polymorphisms, Table S4: Allele frequencies of selected genetic polymorphisms in the Hungarian general and Roma populations; Table S5: Association analysis of selected genetic polymorphisms with alcohol consumption phenotypes in the Hungarian general and Roma populations
