# Supplementary material for: Taste Preference-Related Genetic Polymorphisms Modify Alcohol Consumption Behavior of the Hungarian General and Roma Populations
Source: Genes (Basel). 2023 Mar 7;14(3):666. doi: 10.3390/genes14030666 (PMC10048713; doi:10.3390/genes14030666)
Supplement: Supplementary file 1 [file genes-14-00666-s001.zip › genes-2187833-supplementary.pdf]

Table S1. Demographic characteristics of the two study samples.

Characteristic*	Variable	Hungarian general	Hungarian Roma	p-value**
Marital status n (%)	married	253 (61.71%)	256 (66.15%)	0.240
	single	95 (23.17%)	71 (18.35%)	
	widow, divorced	60 (14.63%)	55 (14.21%)	
Educational level n (%)	primary education or less	86 (20.98%)	327 (84.50%)	**<0.001**
	secondary education	109 (26.59%)	42 (10.85%)	
	high school	138 (33.66%)	12 (3.10%)	
	tertiary education	75 (18.29%)	1 (0.26%)	
Self-perceived financial status n (%)	good	127 (30.98%)	58 (14.99%)	**<0.001**
	satisfactory	227 (55.37%)	207 (53.49%)	
	bad	48 (11.71%)	117 (30.23%)	
Economic activity n (%)	worker	303 (73.90%)	228 (58.91%)	**<0.001**
	inactive***	69 (16.83%)	69 (17.83%)	
	unemployed	32 (7.80%)	83 (21.45%)	

*Unequal to 100% due to missing cases. **Pearson chi-square and Fisher's exact test. Bold indicates statistical significance. ***Inactive: pensioner, other allowance, student.

Table S2. Drinking categories of the Hungarian general and Roma populations according to the 1st three questions of the AUDIT questionnaire

Drinking frequency prevalence (AUDIT question 1)	never	monthly or less	2-4 times a month	2-3 times a week or more	p-value*
**HG n (%)**	191 (46.81%)	116 (28.43%)	49 (12.01%)	52 (12.75%)	**<0.001**
**HR n (%)**	202 (52.60%)	145 (37.76%)	16 (4.17%)	21 (5.47%)	
**Prevalence of number of drinks consumed (AUDIT question 2)**		**1 or 2**	**3 or 4**	**5 or more**	
**HG n (%)**		341 (83.58%)	48 (11.76%)	19 (4.66%)	0.378
**HR n (%)**		303 (79.74%)	55 (14.47%)	22 (5.79%)	
**The prevalence of consuming 6 or more alcohol per occasion (AUDIT question 3)**		**never**	**less than monthly**	**monthly or more offen**	
**HG n(%)**		332 (80.98%)	57 (13.90%)	21 (5.12%)	0.400
**HR n (%)**		321 (84.03%)	41 (10.73%)	20 (5.24%)	

HG: Hungarian general population; HR: Hungarian Roma population. *Pearson chi-square and Fisher's exact test. Bold indicates statistical significance.

Table S3. Hardy-Weinberg equilibrium test for selected genetic polymorphisms

Gene	SNP	Position	Consequence	European allele frequencies*	European genotype frequencies*	HG (p-value of HWE)	HR (p-value of HWE)
TAS1R3	rs307355	chr1:1329774	2KB Upstream Variant	T: 0.079<br>C: 0.921	TT: 0.012<br>CC: 0.855<br>CT: 0.133	0.978	0.996
TAS2R38	rs713598	chr7:141973545	Missense Variant (Ala49Pro)	C: 0.578<br>G: 0.422	CC: 0.338<br>CG: 0.479<br>GG: 0.183	0.021	0.362
TAS2R19	rs10772420	chr12:11021677	Missense Variant (Arg299Cys)	G: 0.495<br>A: 0.505	AA: 0.272<br>AG: 0.465<br>GG: 0.262	0.892	0.667
CA6	rs2274333	chr1:8957145	Missense Variant (Ser90Gly)	A=0.709<br>G=0.291	AA: 0.525<br>AG: 0.368<br>GG: 0.107	0.052	0.949

HG: Hungarian general population; HR: Hungarian Roma population; SNP: single nucleotide polymorphism; HWE: Hardy-Weinberg equilibrium; Hardy-Weinberg equilibrium (HWE) was estimated using the "hwsnp" function in STATA [1]. Significance threshold for Hardy-Weinberg equilibrium was 0.001. *TAS1R3*: Taste receptor type 1 member 3; *TAS2R38*: Taste 2 receptor member 38; *TAS2R19*: Taste receptor type 2 member 19; *CA6*: Carbonic anhydrase VI. *dbSNP

Table S4. Allele frequencies of selected genetic polymorphisms in the Hungarian general and Roma populations

Gene	SNP	Allele	HG allele count (n)	HG allele frequency	HR allele count (n)	HG allele frequency	p-value
TAS1R3	rs307355	C	730	0.90	662	0.91	0.588
		T	80	0.10	66	0.09	
TAS2R38	rs713598	C	446	0.56	433	0.60	0.107
		G	352	0.44	289	0.40	
TAS2R19	rs10772420	G	453	0.56	435	0.60	0.087
		A	357	0.44	287	0.40	
CA6	rs2274333	A	553	0.69	513	0.71	0.418
		G	249	0.31	211	0.29	

HG: Hungarian general population; HR: Hungarian Roma population; SNP: single nucleotide polymorphism; *TAS1R3*: Taste receptor type 1 member 3; *TAS2R38*: Taste 2 receptor member 38; *TAS2R19*: Taste receptor type 2 member 19; *CA6*: Carbonic anhydrase VI; Allele frequencies were estimated by "genhw" program in STATA [2]. To test the differences in the allele frequencies between the two populations the $\chi 2$ test was applied.

Table S5. Association analysis of selected genetic polymorphisms with alcohol consumption phenotypes in the Hungarian general and Roma populations

		HG	HR	HG	HR
**Phenotype (AUDIT question)**	**Gene, SNP**	**Dominant genetic model p-value**	**Dominant genetic model p-value**	**Recessive genetic model p-value**	**Recessive genetic model p-value**
AUDIT1	*TAS1R3* rs307355	0.500	0.289	0.381	0.416
	TAS2R38 rs713598	0.872	0.205	0.359	0.075
	TAS2R19 rs10772420	0.277	0.862	0.260	0.408
	CA6 rs2274333	0.443	0.195	0.861	0.354
AUDIT2	*TAS1R3* rs307355	0.808	0.544	0.637	0.353
	TAS2R38 rs713598	0.402	0.812	**0.028**	0.255
	TAS2R19 rs10772420	0.557	0.525	0.482	0.684
	CA6 rs2274333	0.787	0.494	0.243	0.264
AUDIT3	*TAS1R3* rs307355	0.517	0.204	0.543	0.876
	TAS2R38 rs713598	0.523	0.499	0.994	**0.048**
	TAS2R19 rs10772420	0.246	0.693	0.108	0.770
	CA6 rs2274333	0.482	0.072	0.550	0.363

HG: Hungarian general population; HR: Hungarian Roma population; SNP: single nucleotide polymorphism; *TAS1R3*: Taste receptor type 1 member 3; *TAS2R38*: Taste 2 receptor member 38; *TAS2R19*: Taste receptor type 2 member 19; *CA6*: Carbonic anhydrase VI; AUDIT: Alcohol Use Disorders Identification Test; AUDIT1: How often do you have a drink containing alcohol? (0p: never; 1p: monthly or less; 2p: 2 to 4 times a month; 3p: 2 to 3 times a week; 4p:4 or more times a week); AUDIT2: How many standard drinks containing alcohol do you have on a typical day when drinking? (0p: 1 or 2; 1p: 3 or 4; 2p: 5 or 6; 3p: 7 to 9; 4p:10 or more); AUDIT3: How often do you have six or more drinks on one occasion? (0p: Never; 1p: Less than monthly; 2p: Monthly; 3p: Weekly; 4p: Daily or almost daily); Association analyses were conducted by using STATA's "qtlsnp" command [1]. Models were defined according to minor alleles. Covariates: gender, age, marital status; Nominally significant p values are presented in bold.

References:

1. Cleves, M., Exploratory analysis of single nucleotide polymorphisms (SNP) for quantitative traits. *The Stata Journal* **2005,** *5*, (2), 141–153.
2. Cleves, M. A., Hardy-Weinberg equilibrium tests and allele frequency estimation. *STATA Technical Bulletin.* **1999**, 48, 34-37.
